# Aromatic Fingerprints: VOC Analysis with E-Nose and GC-MS for Rapid Detection of Adulteration in Sesame Oil

**DOI:** 10.3390/s23146294

**Published:** 2023-07-11

**Authors:** Nadia Sadat Aghili, Mansour Rasekh, Hamed Karami, Omid Edriss, Alphus Dan Wilson, Jose Ramos

**Affiliations:** 1 Department of Biosystems Engineering, University of Mohaghegh Ardabili, Ardabil 56199-11367, Iran; aghili_nadia@uma.ac.ir; 2 Department of Petroleum Engineering, Knowledge University, Erbil 44001, Iraq; hamed.wur.nl@gmail.com; 3 Department of Computer, Rafsanjan Branch, Islamic Azad University, Rafsanjan 77181-84483, Iran; eng.omidedriss@gmail.com; 4 Southern Hardwoods Laboratory, Pathology Department, Center for Forest Health & Disturbance, Forest Genetics & Ecosystems Biology, Southern Research Station, USDA Forest Service, 432 Stoneville Road, Stoneville, MS 38776-0227, USA; 5 College of Computing and Engineering, Nova Southeastern University (NSU), 3301 College Avenue, Fort Lauderdale, FL 33314-7796, USA; jr1284@nova.edu

**Keywords:** oil adulteration detection, edible oils, chemometrics, gas sensors, machine learning, mass spectroscopy, electronic nose, volatile organic compounds (VOCs)

## Abstract

Food quality assurance is an important field that directly affects public health. The organoleptic aroma of food is of crucial significance to evaluate and confirm food quality and origin. The volatile organic compound (VOC) emissions (detectable aroma) from foods are unique and provide a basis to predict and evaluate food quality. Soybean and corn oils were added to sesame oil (to simulate adulteration) at four different mixture percentages (25–100%) and then chemically analyzed using an experimental 9-sensor metal oxide semiconducting (MOS) electronic nose (e-nose) and gas chromatography–mass spectroscopy (GC-MS) for comparisons in detecting unadulterated sesame oil controls. GC-MS analysis revealed eleven major VOC components identified within 82–91% of oil samples. Principle component analysis (PCA) and linear detection analysis (LDA) were employed to visualize different levels of adulteration detected by the e-nose. Artificial neural networks (ANNs) and support vector machines (SVMs) were also used for statistical modeling. The sensitivity and specificity obtained for SVM were 0.987 and 0.977, respectively, while these values for the ANN method were 0.949 and 0.953, respectively. E-nose-based technology is a quick and effective method for the detection of sesame oil adulteration due to its simplicity (ease of application), rapid analysis, and accuracy. GC-MS data provided corroborative chemical evidence to show differences in volatile emissions from virgin and adulterated sesame oil samples and the precise VOCs explaining differences in e-nose signature patterns derived from each sample type.

## 1. Introduction

Adulteration and fraud are important issues that have affected the commercial production and marketing of plant-based oils (e.g., olive, avocado, and other high-valued vegetable oils), and they have gained significant attention in the sesame oil industry in recent years [1,2]. Sesame oil (SEO) is an edible oil with high economic and nutritional values and has been extensively employed as a food flavoring with a unique taste [3,4]. The phytochemicals of SEO are considered protective of human health in that they act synergistically as antioxidants, anti-hypertension, anti-mutation, anti-inflammatory, and anti-thrombotic agents, as well as promotors of heart (cardiac) health [5,6].

Food quality reports worldwide have had a major focus on issues relating to the quality and adulteration of edible oils, accounting for 24% (the highest contribution) among the important issues discussed within such reports [7]. Valuable edible oils are, unfortunately, major targets for frequent fraudulent adulteration with cheaper oils due to economic motivations [8,9]. Adulteration is a serious problem, particularly for expensive oils, such as sesame and olive oil. Sesame oil is extensively mixed and diluted with lower-cost oils such as canola oil [10]. Regardless of preventative regulations, some unauthorized producers add low-cost vegetable oils to sesame oil or directly add chemically synthesized sesame essential oil to low-cost oils [11]. Therefore, the quality of sesame oil must be guaranteed in terms of economic market value of the product and health considerations for the benefit of consumers. However, the detection and identification of vegetable oil adulterations are not easy tasks because some of the volatile organic compound (VOC) emission components from low-quality oils are often similar to those of high-quality oils [12].

The detection of sesame oil fraud and adulteration involves the identification and quantification of adulterants, contaminants, or additives that compromise the quality and authenticity of the product. Various analytical techniques and methods have been developed to address this challenge. These methods aim to accurately determine the composition and origin of sesame oil, distinguishing it from adulterated or counterfeit products [1]. One commonly used approach for sesame oil authentication is based on the analysis of fatty acid profiles [13]. Sesame oil has a distinctive fatty acid composition, characterized by the presence of specific fatty acids, such as oleic acid, linoleic acid, and palmitic acid [14]. Gas chromatography (GC) and high-performance liquid chromatography (HPLC) are widely employed to separate and identify fatty acids in oil samples [15,16]. By comparing the fatty acid profiles of suspected samples with known authentic sesame oil profiles, it is possible to identify adulteration or oil substitutions [17]. In addition to fatty acid profiling, advanced techniques, such as nuclear magnetic resonance spectroscopy (NMR), near-infrared spectroscopy (NIRS), and mass spectrometry (MS), have been utilized to enhance the accuracy and reliability of sesame oil authentication [18]. These methods provide detailed information about the chemical composition, molecular structure, and isotopic characteristics of the oil, enabling a more comprehensive analysis of potential fraud due to mixing with lower-quality vegetable oils [19].

Using analytical techniques with chemical sensors for the detection and determination of VOCs is an excellent approach to evaluate the quality of oil samples and to distinguish original high-quality vegetable oils from adulterated, corrupted, or externally spoiled oils [20]. The concentration of VOC emissions from vegetable oils is not necessarily directly related to their impact on the oil aroma and flavor, as it depends on the aroma threshold of each volatile compound, which can vary from 0.017 to 40,000 µg/kg [21]. Various analytical methods have been proposed using high-pressure liquid chromatography (HPLC), gas chromatography (GC), and gas chromatograph–mass spectrometry (GC-MS) to determine the major and minor VOC components of oils [14,22]. The use of expert human sensory panels for analysis is costly and time-consuming. Moreover, the results from human panels cannot be quantified for precise evaluations. There is an important need to develop systems that can detect and quantify the adulteration of low-cost oils in sesame oil samples. Improved detection systems should be portable, low-cost, rapid, non-destructive, and utilize effective chemical sensors to quickly assess adulteration and fraud associated with commercial vegetable oils [10]. Electronic nose (e-nose) devices are particularly well suited for detecting differences in VOC emissions from plant products but are not normally designed to identify individual VOCs present in sample analytes. Metal oxide semiconducting (MOS) gas sensors are the most common type of e-nose sensors used for analyzing VOC emissions from commercial plant products. MOS sensors undergo changes in electrical conductivity when VOCs adsorb to sensor surfaces, which can be measured and correlated with the types and relative amounts of different VOCs present [23]. An electronic nose system typically consists of an array of electronic chemical sensors with VOC-detection specificity and often utilizes a suitable sensor-response pattern recognition system. It is capable of recognizing simple or complex odors [24]. E-nose technologies offer a simple and effective approach for the detection and identification of adulterated vegetable oils. By leveraging the capability of e-noses to analyze VOC emissions associated with product aroma, these gas detection devices can distinguish between authentic and adulterated products [25,26]. Continued research and advancements in this field will contribute to more robust and reliable methods for combating fraud in the sesame oil industry, ensuring consumer trust and product integrity. However, it is important to note that while e-nose technologies show promise, there are still significant challenges because this is still an emerging field with ongoing research and development [16]. Further studies are needed to optimize e-nose sensor arrays, improve detection limits, and enhance the specificity and selectivity of the electronic nose for sesame oil fraud detection.

The objectives of this research were to (1) evaluate the capabilities of electronic nose technology as a rapid, low-cost, practical tool for the detection and discrimination of pure sesame oil from adulterated versions containing soybean and corn oils at different mixture percentages, and (2) to compare e-nose output data with GC-MS analytical data showing chemical differences in vegetable oil sample types. This study aimed to investigate the physical and chemical characteristics of pure sesame oil compared to adulterated mixtures for human health considerations and to preserve the high-cost value of sesame oil by improving quality assurance methods. By employing the electronic nose, the research seeks to enhance capabilities to accurately detect differences between genuine sesame oil and mixed or adulterated oils, ultimately contributing to the prevention of adulteration fraud in the sesame oil industry.

## 2. Materials and Methods

### 2.1. Sample Preparation

Iranian sesame seeds (*Sesamum indicum*), also called benne, of cultivar ‘Yekta’ were used for oil extraction. Yekta is a new sesame cultivar derived from a genetic cross (B5 × M7). The ‘Yekta’ cultivar matures early and is recommended for northern parts of Iran at altitudes >1000 m above sea level. Iranian sesame is considered a good source of natural antioxidants for medicinal and commercial uses. After preparing the sesame seeds, oil was rendered from the seeds using a mechanical pressure device, a BD 65 Oil Cold Press Machine (Bekrdaneh company, Esfahan, Iran), followed by several steps of seed oil filtration, performed to remove solid impurities and contaminants. Samples of pure, genuine sesame oil and mixed adulterated sesame oil (containing different percentages of soybean and corn oil additives) were evaluated in this research. Four adulteration levels (25%, 50%, 75%, and 100% or pure adulterant oil) were analyzed and evaluated for each of the two types of added adulterant oil, resulting in a total of 9 separate treatments (oil sample types) with 15 replications of each sample type prepared for each treatment. Fifteen separate glass vials containing 100 mL of each oil sample type, consisting of pure sesame oil and the four adulterated versions, were prepared as replications for e-nose analysis. Nine additional vials containing 100 mL of each oil sample type were prepared and used for GC-MS testing.

### 2.2. Olfactory Machine (Electronic Nose)

**Figure 1:**
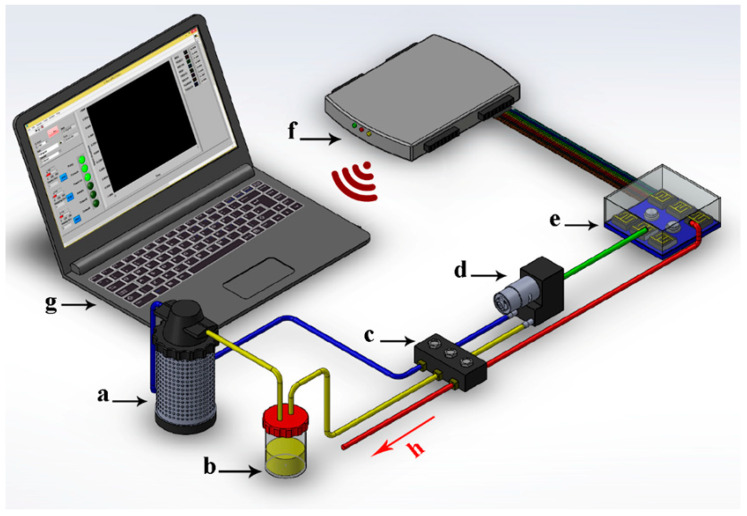
Schematic air flow diagram of the e-nose aroma system, including the following hardware components: (**a**) activated carbon filter, (**b**) sample, (**c**) valve, (**d**) pump, (**e**) sensor array, (**f**) data card, (**g**) computer, and (**h**) air outlet (red arrow).

The olfactory machine system used in this research was developed and constructed by Karami, Rasekh, and Mirzaee-Ghaleh, as described previously [9]. The names of individual metal oxide semiconductor (MOS) sensors within the 9-sensor array are (in order, with the primary VOCs and gases detected) as follows: MQ3 (alcohols), TGS822 (organic solvents), MQ136 (sulfur dioxide), MQ9 (carbon monoxide and combustible gases), TGS813 (aliphatic alkanes), MQ135 (ammonia, benzene, sulfides), MQ8 (hydrogen), TGS2620 (alcohols, organic solvents), and MQ4 (methane, natural gas). This analytical system is divided into two working components: (1) hardware consisting of a MOS e-nose device with associated components, and (2) software required to operate the machine and for data analysis. The hardware components include a data collection card, active carbon air filter, array of nine MOS sensors, computer, power supply, sampling chamber, diaphragm air pump (model R385 with a flow rate of 1.5 L/Min), three electronic two-way diaphragm valves, accessories, and air tube connections (Figure 1). Oil samples for analysis were placed into the sample chamber and left to build VOC headspace for 15 min prior to sample analysis. Clean, filtered room air was passed over the sensors for 150 s to clean the sensors and sensor chamber before sampling and to give time for the sensors to reach baseline response levels for each sensor. The headspace VOCs were injected into the sensor chamber at a gas air flow rate of 200 mL/min via a pump. The output voltage of each sensor changed in response to VOC adsorption to sensor surfaces (the basis of VOC detection), depending on sensor sensitivity to individual VOC components of the headspace. The voltage response of the sensors was recorded using the data card at 1 s intervals. The final step, following sample analysis runs, involved air passing again over the sensors for 200 s to purge the previous sample, clean the sensor surfaces, and to allow the analyzed sample inside the sample chamber to be discharged through a pump. The device was then prepared for the next sample. After sampling, the nine-sensor output responses were preprocessed in different ways to optimize the output signals from the sensor array and to increase the efficiency of the available information. The extraction data obtained from sensor-response output signals were obtained relative to sensor baseline values, based on the sensor output readings at the air-injection point [27].

### 2.3. Data Preprocessing Prior to Feature Extraction 

Data preprocessing was required before extraction of data features to reveal the relative responses of sensors and to increase their accuracy in the pattern detection analysis [28]. The data obtained from gas sensors usually have fluctuations or noise. Preprocessing prepares sensor responses for subsequent statistical analysis, including the extraction of signal features, by improving and optimizing sensor data [29]. Data cleaning, data integration, data reduction, and data transformation are the most important activities in the data preprocessing section. The processing of sensor signals involves three general stages, including compression, baseline correction, and normalization. Baseline correction is conducted to compensate for noise and drift and to increase the response quality of the sensors. Conventional methods of preprocessing gas sensor data include relative, differential, or fractional methods. A fractional method was utilized in this study for preprocessing (normalizing) sensor output signals based on application of the normalized signal equation (Equation (1)):

(1) $$Ys(t)=\frac{Xs(t)-Xs(0)}{Xs(0)}$$

in which *Ys*(*t*) is the normalized signal, *Xs*(*t*) denotes the output signal of the sensor due the presence of volatile substances (VOC), and *Xs*(0) shows the base signal of the sensors or baseline [9].

### 2.4. Data Analysis Method

Chemometrics is a branch of chemistry which utilizes mathematics, statistics, and logic to obtain further information on the chemical systems, design, and selection of optimal empirical processes and provide optimal chemical data [30]. Chemometrics can be utilized in process controls, pattern detection analysis, optimization, and symptom processing. Principal component analysis (PCA), linear differential analysis (LDA), support vector machine (SVM), and artificial neural network (ANN) were employed for data modeling and statistical analysis in this study.

PCA is an unsupervised multi-variable statistical analysis method for linear data compression, decreasing the data dimension, and feature extraction. PCA provides the possibility to detect outliers, pattern recognition in the sample distribution, and inter-variable relationships and classes [31]. This method has been extensively utilized for displaying the response of an olfactory machine to simple and complex aromas to offer qualitative information for pattern recognition [32]. LDA is a supervised classification approach, which provides linear conversion of n-dimensional vectors (number of samples) to an m-dimensional space (number of variables, m < n) [33]. Support vector machines (SVMs) are a popular effective supervised classifier in data mining and pattern recognition. SVMs are useful for handling both linear and nonlinear data. This method transforms input data into a higher-dimensional feature space using kernel functions. In this transformed space, SVMs seek to find the optimal hyperplane that maximally separates the different classes, thus enabling accurate classification [34].

ANN is a method classified in the artificial intelligence class of computer science with fundamental differences from other computational methods. Multi-layer perceptron neural network is a feed-forward network, which can be utilized in chemometrics analysis to solve pattern recognition and prediction problems using either supervised or unsupervised methods [35,36].

### 2.5. Chemical Analysis of Oil Samples

A gas chromatograph used in tandem with a mass spectrometer (GC-MS) with a 30 m long HP-5 column, having an internal diameter of 0.250 mm, was utilized to identify the VOC chemical parameters of the vegetable oils. This device was set up for injecting liquid samples with split/splitless inlet dilution. Mass spectrometer detector (MSD) analysis was employed for both qualitative and quantitative determinations. The detector was equipped with an EI-type ionization system with a single quadrupole MS analyzer. An EMP triple-axis detector with very low noise and drift was employed to achieve high sensitivity.

All oil samples were prepared as methyl ester derivatives of fatty acids using the Savage and McNeil (1998) method [37], slightly modified by placing 10 mg of oil dissolved in 0.5 mL of hexane in a test tube, followed by adding 2 mL of 0.01% (*w*/*v*) NaOH, prepared in dry methanol. The test tube was kept in a water bath for 10 min. A 3 mL aliquot of BF3 reagent was added to the mixture kept in the warm-water bath at 60 °C for an additional 10 min. After the reaction, the test tube was placed in a cold-water bath at 1 °C, and 2 mL of 20% NaCl with 1 mL of hexane were added and stirred to a uniform mixture. The resulting mixture was centrifuged at 4000 rpm for 10 min, and the hexane layer containing methyl ester of fatty acids was separated for subsequent GC-MS analysis [1].

### 2.6. Model Evaluation Metrics

Five evaluation criteria (Accuracy, Precision, Recall, Specificity, AUC) were used to assess the performance of the statistical models. AUC is the area under the ROC curve that represents the tradeoff between Recall and Specificity. Four parameters of true positive (TP), false positive (FP), true negative (TN), and false negative (FN) were used to calculate the evaluation criteria. Accuracy was determined based on the ratio of correctly classified samples (TP and TN samples) to the total number of samples. Sensitivity was indicated by the ratio of TP samples to the total number of positive samples (TP and FN samples). Precision was determined by the ratio of TP samples to the total number of positive predictions (TP and FP samples). F1 score was determined as the harmonic mean of precision and sensitivity [38].

## 3. Results

### 3.1. GC-MS Results

The GC-MS analyses of sesame oil (with and without adulterations with lower-cost oils) were compared to published information on the major fatty acid composition of these individual vegetable oils as follows. Sesame oil primarily contains linoleic acid (C18:2, ω-6) and oleic acid (C18:1, ω-9), accounting for 80% of the total fatty acids present [4]. Corn oil contains 65.5% linoleic acid as well as other bioactive compounds, including sterols (β-sitosterol, campesterol and stigmasterol), phenolic acids, and flavonoids [39]. Soybean oil contains 51% linoleic acid (polyunsaturated), 7–10% alpha-linoleic acid (polyunsaturated), 23% oleic acid (monounsaturated), 4% stearic acid (saturated), and 10% palmitic acid (saturated). The high linoleic acid content in soybean oil makes it unsuitable as a frying oil [40].

GC-MS analysis revealed eleven major compounds representing about 82–91% of the oil, as listed in Table 1. The main components of sesame oil, distinguishing it from corn and soybean oils, include linoleic acid (63.241%), palmitic acid (17.918%), and stearic acid (5.516%). Stearic acid (53.474%) and palmitic acid (26.805%) are the two main components of soybean oil and, similarly, corn oil contains stearic acid (56.412%) and palmitic acid (23.454%) as the two main constituents. Because these major compounds are present in all three oils, these VOCs are not suitable chemical targets for detecting adulteration in sesame oil. Consequently, we found some unique VOCs to distinguish between volatile emissions from each oil type using the electronic nose. Capric acid (0.007%) was the first compound we discovered that specifically indicated adulteration of sesame oil by soybean oil because sesame oil contains no capric acid. Therefore, any level of this compound in sesame oil indicates adulteration. The amount of this compound reached 0.002% in SB3 oil. For corn oil, this detection of adulteration of sesame oil was more difficult based on chemical compositions because both oils have a similar composition, although corn oil does not contain gondoic acid and oleic acid. Thus, the small amount of these two compounds in sesame oil (approximately 1%) is diluted to lower levels when mixed with other oils, but this minor difference makes it difficult to detect adulteration based on these minor components alone [41].

**Figure 2:**
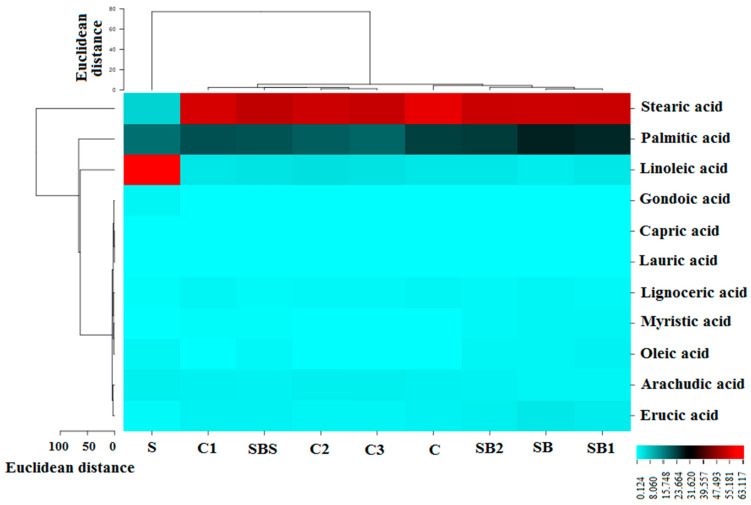
Heat map indicating differences in volatile compound emissions from different oil groups. Volatile compounds were plotted on the Y-axis, while the X-axis corresponds to different oil type groups. Oil-type abbreviations: Pure sesame Oil (S), Soybean oil 100% (SB), 75% Soybean + 25% Sesame (SB1), 50% Soybean + 50% Sesame (SB2), 25% Soybean + 75% Sesame (SB3), Corn oil 100% (C), 75% Corn + 25% Sesame (C1), 50% Corn + 50% Sesame (C2), and 25% Corn + 75% Sesame (C3).

Cluster analysis was conducted using GC-MS fatty acid data to classify and detect adulteration in sesame oil. The results were plotted in a heat map (Figure 2). The heat map provides an alternative visual representation of differences between high-dimensional data by using Euclidean distances to indicate statistical differences between fatty acid VOCs (chemical-based composition) of cluster groups. The cluster lines on the left side of Figure 2 represent clusters of VOCs. Volatile compounds were grouped into two main clusters. Stearic acid had the highest level in all three vegetable oil sample types and was placed in the first cluster. The second cluster was divided into three sub-branches; palmitic acid and linoleic acid were placed in the first two groups, while other volatile fatty acid compounds were placed in the third group with the lowest content. The clustered lines at the top of Figure 2 represent different oil groups. The three different groups of oils were divided into two main clusters. Pure sesame oil was placed in the first cluster on the left side of Figure 2, while the other two oil groups were placed in the second cluster. Using this heat map, pure sesame oil could be easily distinguished from the other oil groups.

Euclidean distance (ED) measures showed the level of chemical relatedness of VOC emission components (quantitative VOC content) from each oil sample type (based on the presence of individual fatty acid VOCs), providing indications of chemical differences between oil samples based on specific volatile fatty oils detected using GC-MS analysis. Notice that pure sesame oil contained very little stearic acid (major fatty acid component) and also low levels of the other eight minor fatty acid components (gondoic acid–erucic acid) at the bottom of the heat map (ED values < 8.0, indicated by cyan blue color in heat map) compared to all other pure oil types and sesame mixtures containing added soybean or corn adulterants that did contain the stearic acid as a major VOC component (ED value range 40–55, indicated in dark red). However, pure sesame oil did share the presence of high levels of palmitic acid with all other pure oil types and adulterated sesame oil mixture types (ED value range 22–30, indicated in dark gray). Pure sesame oil was the only oil sample type that contained high levels of linoleic acid (ED value > 60, indicated in bright red).

### 3.2. E-Nose Analysis Combined with PCA and LDA

**Figure 3:**
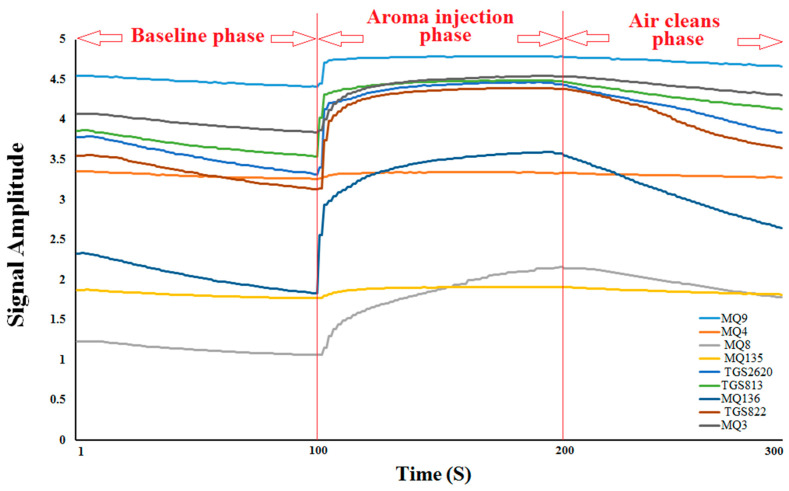
Aroma signatures resulting from 9 gas sensor-output responses of the e-nose system to aroma VOC emissions of oil samples.

The unique aroma signatures, based on the maximum sensor outputs recorded during an e-nose analytical run (also known as smellprint or aroma signature patterns) derived from the nine-sensor MOS gas sensor array, are presented in Figure 3. These smellprint patterns represent the total multisensory-array output response to individual volatile organic compound (VOC) emissions from the oil samples. These sensor response patterns are visual representations of the distinctive sensor array responses to different aroma VOCs emitted by each distinct oil sample type. Data from the aroma injection phase of the e-nose analysis runs were used for sample analyses.

**Figure 4:**
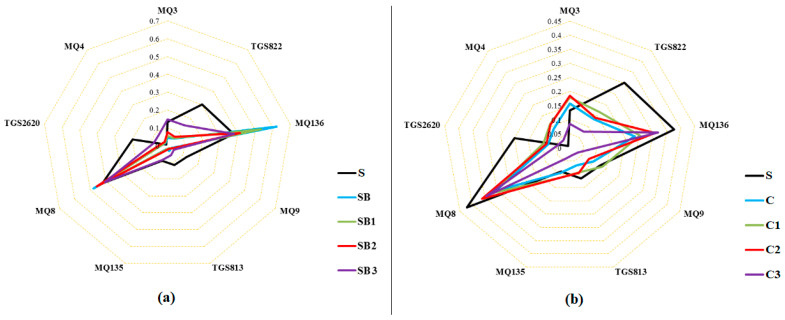
Radar plot of sensor responses to adulterated mixtures of vegetable oil VOCs. (**a**) Sensor response plot for sesame oil mixed with soybean oil, and (**b**) Sensor response plot for sesame oil mixed with corn oil. Oil-type abbreviations: Pure sesame Oil (S), Soybean oil 100% (SB), 75% Soybean + 25% Sesame (SB1), 50% Soybean + 50% Sesame (SB2), 25% Soybean + 75% Sesame (SB3), Corn oil 100% (C), 75% Corn + 25% Sesame (C1), 50% Corn + 50% Sesame (C2), and 25% Corn + 75% Sesame (C3).

Radar plots, displaying individual sensor output responses (composing the smellprint patterns) from the e-nose sensor array, provided indications of relative differences in sensor intensities to different vegetable oil VOC components (Figure 4). Mean sensor e-nose output data from each oil class resulted in different patterns of sensor responses that reflected chemical differences in fatty acid and other VOC components present in the volatile emissions from sesame oil adulterated with different lower-valued oils. The highest sensor response in all oil groups was obtained from e-nose sensors MQ136 and MQ8. Sensors TGS822 and TGS2620 exhibited the highest response in oil samples of sesame alone, with the highest sensor response obtained from sensor MQ3. The rest of the sensors responded to the sample aroma at lower intensities. Therefore, differences in the aroma characteristics of each sample were indicated by different sensor output intensity response patterns from the e-nose sensor array.

**Figure 5:**
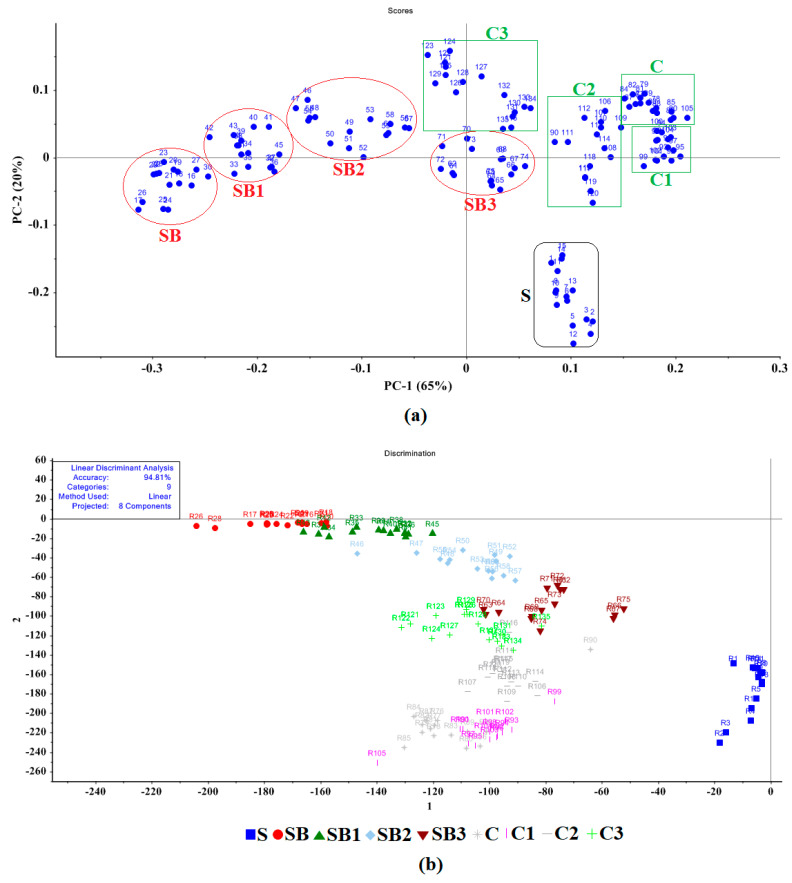
E-nose data plots for sesame oil sample types, based on statistical models, including: (**a**) PCA results, and (**b**) LDA results for different levels of sesame oil adulteration. Oil-type abbreviations: Pure sesame Oil (S), Soybean oil 100% (SB), 75% Soybean + 25% Sesame (SB1), 50% Soybean + 50% Sesame (SB2), 25% Soybean + 75% Sesame (SB3), Corn oil 100% (C), 75% Corn + 25% Sesame (C1), 50% Corn + 50% Sesame (C2), and 25% Corn + 75% Sesame (C3).

PCA indicated differences between oil sample types based on the recognition of pattern differences in e-nose sensor output data responses, in which the higher the PCA total variance, the greater the discrimination between sample types (Figure 5). The values of stable sensor response to different oil samples were utilized for PCA analysis after normalization. Figure 4a shows the contribution of PC1 and PC2, accounting for 65% and 20%, respectively, of the total variance (85%) from these two principal components. As seen, the pure sesame oil (S) was completely differentiated, as appeared in the fourth quarter of PCA of other oil groups. The soybean (SB) and corn (C) oils were also placed on the left and right sides of the diagram, respectively.

The LDA method was utilized to reduce the classification differences and enhance discrimination between different oil groups based on e-nose sensor responses. The variance in the total samples for the LDA method was 94.81 according to the LDA results presented in Figure 4b. For the PCA method, the pure sesame oil (S) sample was completely differentiated from the other oils. Some overlaps can be detected between the other oil groups, such that one SB oil sample was mistakenly related to the SB1 group. Three oil groups (C, C1, C2) overlapped considerably in the data plots.

### 3.3. Machine Learning Classification

Pattern recognition methods are a valuable alternative for data cluster separation, but this task becomes more difficult in systems with large amounts of data and overlapping data plot regions. Therefore, machine learning algorithms are more effective for independent sample classification to optimize and automate data processing, reduce subjectivity, and increase efficiency in predicting results and method robustness [9,21]. SVM and ANN classifiers were used in this analysis. The outputs of nine sensors were regarded as inputs of the model, while the number of oil groups (9 groups) was taken as the model output. Of the total data, 70, 15, and 15% were used for training, validation, and testing, respectively.

SVM was used as a supervised pattern recognition method to detect adulteration in pure sesame (S) oil from SB- and C-type oil-adulterated samples. Two types of statistical models, based on SVM methods (C-SVM and Nu-SVM), were used. Nu, C, and γ parameters were validated by trial and error and through error minimization. Four types of linear, polynomial, radial base, and sigmoid kernels were applied to classify pure S-type oil from SB- and C-type oil mixtures. The performance of SVM models was evaluated based on classification accuracy (Table 2). The highest accuracy was observed in the C-SVM method with a linear kernel function. The classification accuracy for training and validation was 97.18 and 93.33%, respectively, exhibiting the highest accuracy in the classification of different oil sample types.

ANN results are also depicted in Table 3. The models were evaluated in terms of the percentage of correct diagnosis (CCR) and root mean square error (RMSE). According to the results obtained for nine different oil groups (sample types), 9-10-9 topology exhibited the best results, with respective R^2^ values of 0.956 and 0.936 for training and test analyses. The RMSE values were 0.001 and 0.018 for training and validation tests, respectively. The model had a total recognition accuracy of 95.6%. Regarding the lower performance value for the training phase (compared to the testing phase), there were no signs of under- or overfitting.

**Figure 6:**
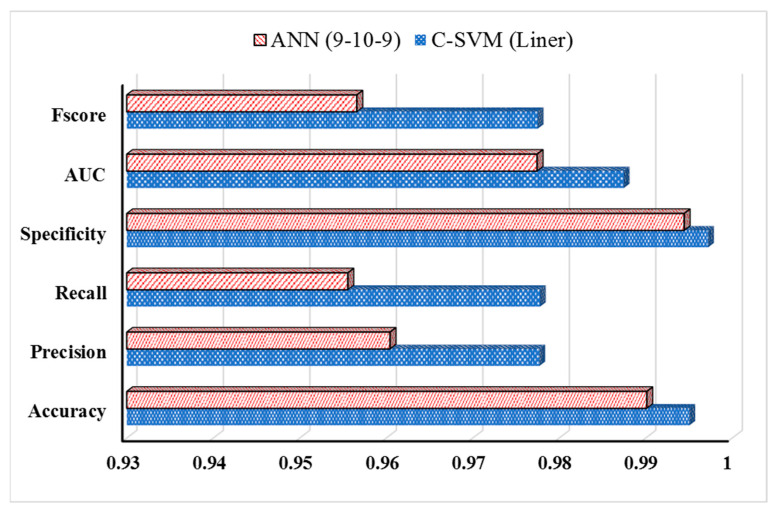
Performance parameters of ANN and SVM statistical models in the detection of adulteration in sesame oil.

The disturbance matrix and functional parameters of the ANN and C-SVM models are compared in Figure 6. The results obtained from an average of 135 data points from the analyzed oil samples indicated that only three samples were misclassified by the SVM method, while the ANN method misclassified six samples. Sensitivity and specificity parameters are another method to evaluate the ability of the electronic nose to distinguish between purse sesame oil (S) from SB and C adulterated oil mixture types. Sensitivity is the number of true-positive samples, while specificity implies the rate of correctly identified true negatives. The sensitivity and specificity of SVM and ANN methods were 0.978, 0.956, 0.949, and 0.960, respectively. Performance parameters of SVM and ANN methods are compared using bar graphs in Figure 5. The C-SVM method, a linear model function, provided better classification and discrimination between oil sample types than the ANN method.

## 4. Discussion

E-nose fatty acid VOC analysis data, derived from sensor responses to oil sample headspace volatiles, combined with statistical methods for data analysis, yielded an effective means of distinguishing between pure sesame oil and two other different vegetable oil types (soybean and corn) in various adulterated mixtures with sesame oils in the current study. A comparison of e-nose data analysis results, using four separate statistical models to determine their effectiveness in discriminating between pure sesame oil and other adulterated mixtures of this oil, was based on e-nose analysis of VOC emissions that consisted primarily of volatile fatty acids present in the headspace of each oil sample type. GC-MS data provided the analytical chemical support data to show the basis for chemical differences in VOC emissions (from different oil sample types), which backed up and confirmed related differences revealed from e-nose analysis of the different sample types revealed from unique e-nose smellprint signatures recorded for pure vs. adulterated sesame oil samples from the sensor array.

Combining e-nose and statistical models for comparisons of volatile compound emissions from oil samples provided a qualitative means for assessing oil quality through the detection of adulteration, oil product spoilage, or deterioration by oxidation and other external factors, necessary for confirming the authentication of oil purity and quality to avoid product fraud. GC-MS data indicated that it was often difficult to detect the adulteration of sesame oil with both soybean and corn oil because the fatty acid composition of these oils is very similar with high amounts of palmitic acid (major VOC components), but pure sesame oil is very low in stearic acid content. However, the presence of much higher levels of linoleic in pure sesame oil than in the other, lower-quality soybean and corn oil types provided another major discovered difference in VOC emissions. These two major VOC emission differences between pure sesame oil and other oil types and mixtures made it possible to effectively detect these VOC differences in e-nose sensor-array response patterns, indicative of adulteration obtained through statistical analysis of sensory data derived from the MOS electronic nose. The combination approach of using an MOS e-nose with appropriate statistical modeling provided results indicating that this is a promising approach for detecting fraudulent adulterations of sesame oil. PCA explained 96% of the total variance (with two principal components) in e-nose data when comparing pure sesame oil with other oils and sesame oil mixtures. However, the SVM linear model method, along with the Nu-SVM method, showed the highest classification accuracies. Based on LDA and QDA statistical methods, the oil classification accuracy was 94.07 and 100%, respectively. The best discrimination occurred while using 9-10-9 topology that yielded a 95.6% correct classification rate for this neural network method.

Our results for sesame oil e-nose analyses and GC-MS are compared here with published results in the following discussions, which describe other study results that have used GC-MS and/or e-nose devices to analyze different types of vegetable oils for various product quality and purity-certification applications. These studies showed how the methods used in oil production are important in determining final product quality.

Zunin et al. [42] used a quadrupole GC-MS alone with PCA and SIMCA statistical methods to distinguish and classify 105 types of extra virgin olive oil from various Mediterranean regions. Their results showed that 93.4% of the olive oil samples were correctly classified primarily based on differences in terpenoid hydrocarbons, with 90.5% correctly evaluated using a mutual validation method and 80.0% of samples in external tests. They found that oil extraction and production temperature of the headspace were important for assuring product quality. For example, they found that high oil extraction temperatures may accelerate oxidation and enhance decomposition, resulting in off flavors for Ligurian and other Mediterranean olive oils.

Bougrini et al. [43] used an MOS e-nose system based on a tin-oxide five-sensor array and an electronic tongue composed of seven voltametric electrodes to determine the adulterant percentage of sunflower oil in argan oil. PCA results and data envelopment analysis (DEA) showed acceptable results of e-nose data in distinguishing pure argan oil from adulterated mixtures of this oil. Using the SVM method, accuracies of 91.67% and 83.34% were obtained for edible and cosmetic argan oil, respectively. They emphasized the effectiveness of the e-nose in detecting adulteration in argan oil using different contents of sunflower oil (10 to 70%).

Lerma-García et al. [44] also used an MOS electronic nose (Italian EOS 507, Sacmi Imola, SC) with six sensors to classify original oil (with or without phenolic compounds) and to determine the oxidation state that was well correlated with sensorial analyses. Xu et al. [45] employed an e-nose to qualitatively analyze the oxidation of edible oil at accuracies of 98.9%, 95.8%, and 100% in tests differentiating oxidized oils from non-oxidized ones using PCA, CA, and LDA statistical methods, respectively. The LDA results were slightly better than PCA and CA methods. Both studies indicate that LDA and PCA methods show promising accuracy in their respective applications, with LDA consistently achieving high accuracy in both adulteration detection and oxidation analysis.

Cerrato Oliveros et al. [46] employed a 12-sensor FOX-3000 MOS e-nose using LDA, QDA, and artificial intelligence network (ANN) statistical methods to effectively detect the adulteration of extra virgin olive oil with cheaper sunflower oil. Feature methods were selected for a set of desirable specific variables (among treatment types) before supervised pattern recognition methods were applied. The best adulteration detection results were achieved for olive oil (correctly predicting adulteration above 95 and 85% in separate tests) using LDA and QDA, whereas the ANN prediction was slightly lower, and partial least squares (PLS) yielded only 73% detection. The effectiveness of adulteration detection decreased as the percentage of adulterant added decreased. They were also capable of identifying the type of oil used in adulteration, indicating that the methods could be used to classify samples as a function of adulteration percentage.

A relatively recent review article by Roy and Yadav [47] compared the results of e-nose-based studies to detect adulteration in vegetable oils. Among the studies compared in this review, PCA, LDA, and PLS methods for e-nose data were found to be the most suitable for classification and pattern recognition to distinguish the adulteration in edible vegetable oils. These studies collectively suggested the potential use of e-noses coupled with multivariate analysis techniques for fraud detection in various edible plant oils, with details indicated in the following discussions.

Men et al. [48] analyzed soybean oil adulteration at nine different concentrations using an eight-MOS sensor e-nose with PCA and PLS classification and pattern recognition methods. PCA explained 87.2% of the data variance for e-nose classification of adulterated soybean oil. The 50% adulteration level was well separated only by using the PCA method. The PLS model differentiated old frying oil from soybean oil. A 0.843 correlation coefficient and 12.1% error rate for e-nose data showed PLS as an effective model to predict the adulteration in soybean oil. However, the fusion of data from an e-nose and e-tongue was more effective using PCA and PLS methods for adulteration detection in soybeans.

Marina et al. [49] investigated the capability of a surface acoustic wave (SAW) sensor-based e-nose for the classification and detection of virgin coconut oil adulterated using PCA and PLS discrimination methods. They used adulteration blends (*w*/*w*) of 1–10% in increments of 1% and in a range of 10–20% adulteration with 5% increments. Headspace volatiles from oil samples were analyzed, and the best separation of adulterated samples was achieved using PCA, especially when the level of adulteration increased, contributing to 91% accuracy in the data. The PLS study results yielded a coefficient of determination (R^2^) value of 0.91, indicating the potential utilization of the e-nose in the adulteration detection of virgin coconut oil with palm kernel olein oil.

Hong et al. [50] analyzed GC-MS data using PCA (PC1 98.76% and PC2 0.57%), which could discriminate the adulterated samples of palm olein oil by palm stearin oil in a range of 10% to 90% proportions but was not effectively classified below 10% adulterated ratios, although these lower adulteration levels could be well discriminated using DFA (DF1 0.997 and DF2 0.966). The chemical composition of VOC emissions from palm oil was confirmed using a quadrupole mass spectrometer. Another similar study by Man et al. [51], with palm olein oil adulterated with lard (mixing proportions as low as 1%), showed that adulteration could be identified using an e-nose method with Pearson’s correlation coefficient accuracy > 0.90.

The adulteration of peony seed oil with corn oil, rapeseed oil, sunflower oil, and soybean oil was investigated in a study by Wei et al. [52] using the PEN3 e-nose with a selected array of a 10-MOS sensor array in combination with PCA and LDA chemometric statistical methods. PCA analysis well differentiated peony seed oil from all four tested adulterants at an accuracy of 96.7% from e-nose data. There was some partial overlapping within the four adulterant oils and pattern recognition via LDA provided effective discrimination of most oil adulteration types, except for a small overlap of corn oil and rapeseed oil, providing a high level of discrimination of pure peony seed oil from adulteration with cheaper-oil adulterants.

## 5. Conclusions

The electronic nose and GC-MS chemical analysis methods (used in combination) were evaluated in this study for the capability of detecting the adulteration of sesame oil due to amendments with soybean and corn oils, added and mixed at different ratios and concentrations. We propose that the e-nose-based method alone is a simple and low-cost approach to quickly detect adulteration in sesame oil. This method may be utilized as a complementary technique to the quality control and assurance of sesame oil purity. Additional investigations are needed to further optimize experimental parameters and data collection to improve this technique and to fully validate the proposed method for differentiating between pure sesame oil from adulterated mixtures with lower-quality oils at different concentrations. Once e-nose analysis discriminations are confirmed using GC-MS chemical analysis data, e-nose analysis can be accomplished alone without supporting chemical analysis data. The advantages of using e-nose analysis are that it is much faster, cheaper, easier, and more effective in the discrimination and identification of vegetable oil sample types, compared to GC-MS analysis that requires considerably higher expense, more laborious interpretation of chemical data, and much longer delays in obtaining analysis results.

Different methods used with various e-nose instruments described in the literature for various applications related to oil quality control, including detecting adulteration, determining regional origin, and evaluating the impact of production factors on the final product, have demonstrated the versatility of e-nose technologies for oil quality assessments. The choice of the specific e-nose method used depends on the desired objectives and the characteristics of the oils being analyzed. The emergence of e-nose instruments as valuable tools for oil quality analysis has revolutionized the detection of adulteration in vegetable oils. By employing electronic chemical sensors and pattern recognition systems, e-noses have the capability to analyze VOCs emitted by various oil samples. This technology presents a rapid and non-destructive approach for evaluating the authenticity and quality of oils, including the identification of adulteration. The application of an e-nose in the oil industry has demonstrated promising outcomes in distinguishing between unadulterated and adulterated oils. E-nose instruments can effectively detect subtle discrepancies in VOC profiles, enabling the identification of unauthorized substances added to quality oils or the presence of lower-quality oils mixed with higher-quality ones. Through the utilization of statistical models and machine learning algorithms, the e-nose can offer the precise and reliable classification of oil samples, guaranteeing consumer protection and preventing fraudulent practices. E-nose methods are easier to use (simplicity of operation), provide greater speed in terms of analytical results at lower costs, and have the capabilities to handle intricate odor profiles. They serve as a complementary technique to traditional analytical methods, such as GC-MS, by facilitating real-time and on-site monitoring, thereby reducing the reliance on expensive and time-consuming laboratory testing. Moreover, the e-nose contributes to the assessment of oil freshness, stability, and oxidation levels, all of which are important factors in determining overall oil quality. The implementation of e-nose technologies provides an effective solution to inadequate conventional methods for the detection of oil adulteration and the assurance of product integrity. The capacity of e-nose instruments to nondestructively analyze VOC emissions from oils makes them effective and efficient tools for assuring post-harvest quality control in oil products to combat adulteration fraud and safeguard consumer interests.

## Figures and Tables

**Table 1:** Fatty acid chemical composition (%) of vegetable oil VOCs based on GC-MS analysis.

Fatty Acids	Common Name	RT (min)	Vegetable Oil Type *								
			S ^1^	SB	SB1	SB2	SB3	C	C1	C2	C3
Decanoic acid	Capric acid (C10:0)	17.5	0	0.007	0.007	0.009	0.002	0	0	0	0
Dodecanoic acid	Lauric acid (C12:0)	24	0.006	0.202	0.189	0.138	0.056	0.002	0.002	0.011	0.005
Tetradecanoic acid	Myristic acid (C14:0)	29	0.074	1.188	1.076	0.83	0.394	0.239	0.28	0.127	0.192
Hexadecanoic acid	Palmitic acid (C16:0)	33.5	17.918	26.805	26.298	24.028	21.112	23.454	21.364	19.872	18.882
8,11-Octadecadienoic acid	Linoleic acid (C18:2)	36.5	**63.241**	2.407	3.058	3.133	3.35	3.156	3.184	3.759	3.69
9-Octadecenoic acid	Oleic acid (C18:1)	36.9	1.02	1.02	1.274	1.023	0.936	0	0	0	0
Octadecanoic acid	Stearic acid (C18:0)	38.2	**5.516**	53.474	53.218	53.12	52.146	56.412	54.458	53.372	52.757
11-Eicosenoic acid	Gondoic acid (C20:1)	39.5	1.035	0	0	0	0	0	0	0	0
Eicosanoic acid	Arachudic acid (C20:0)	40	2.045	1.185	1.136	1.244	1.349	1.719	1.525	1.817	2.009
Docosanoic acid	Erucic acid (C22:0)	42.5	0.588	2.815	2.331	2.07	1.313	1.271	1.411	0.992	1.184
Tetracosanoic acid,	Lignoceric acid (C24:0)	44.8	0.359	1.049	0.889	0.848	0.623	1.081	1.238	0.886	0.802
Total%			91.802	91.721	91.106	88.361	83.745	89.497	86.128	83.134	82.526
Other compounds			7.198	8.279	8.894	11.639	16.255	10.503	13.872	16.866	17.474

* Vegetable oil type abbreviations: Pure sesame Oil (S), Soybean oil 100% (SB), 75% Soybean + 25% Sesame (SB1), 50% Soybean + 50% Sesame (SB2), 25% Soybean + 75% Sesame (SB3), Corn oil 100% (C), 75% Corn + 25% Sesame (C1), 50% Corn + 50% Sesame (C2), and 25% Corn + 75% Sesame (C3). ^1^ Major VOC-components of pure sesame oil (S) most useful in distinguishing this virgin oil from other oils and adulterated mixtures (indicated by values in bold).

**Table 2:** Results and comparison of Nu-SVM and C-SVM models subjected to kernel functions.

Kernel Function	C-SVM					Nu-SVM			
	C	γ	Training	Validation		nu	γ	Training	Validation
Linear **	**100**	**1**	**97.18**	**93.33**		0.5	1	96.78	93.07
Polynomial	10	1	80.74	77.04		0.5	1	88.15	83.70
Radial basis function	100	0.1	96.48	92.07		0.5	0.01	94.78	92.33
sigmoid	100	0.1	95.55	88.15		0.745	0.01	92.59	91.11

** The values in bold indicate that the C-SVM model (a linear kernel function) showed the best performance in detecting sesame oil adulteration.

**Table 3:** Artificial neural network results.

Topology *	Training		Test		CCR (%) **
	RMSE	R^2^	RMSE	R^2^	
9-5-9	0.074	0.844	0.088	0.823	84.4
9-6-9	0.046	0.899	0.059	0.849	88.3
9-7-9	0.054	0.901	0.065	0.878	89.5
9-8-9	0.035	0.930	0.039	0.901	91.7
9-9-9	0.027	0.928	0.042	0.911	92.6
9-10-9	0.001	0.956	0.018	0.930	95.6
9-11-9	0.038	0.925	0.423	0.915	92

* Topology: A network topology notation refers to the arrangement of a network with its nodes and connecting lines. ** The value of the statistical index of the correct classification rate (CCR) obtained from the confusion matrix.

## Data Availability

Supporting data are not available in publicly archived datasets due to government policy restrictions.

## References

[1] Aghili N.S., Rasekh M., Karami H., Azizi V., Gancarz M. (2022). Detection of fraud in sesame oil with the help of artificial intelligence combined with chemometrics methods and chemical compounds characterization by gas chromatography–mass spectrometry. LWT.

[2] Hai Z., Wang J. (2006). Electronic nose and data analysis for detection of maize oil adulteration in sesame oil. Sens. Actuators B Chem..

[3] Jayaraj P., Narasimhulu C.A., Rajagopalan S., Parthasarathy S., Desikan R. (2020). Sesamol: A powerful functional food ingredient from sesame oil for cardioprotection. Food Funct..

[4] Wan Y., Li H., Fu G., Chen X., Chen F., Xie M. (2015). The relationship of antioxidant components and antioxidant activity of sesame seed oil. J. Sci. Food Agric..

[5] Aslam F., Iqbal S., Nasir M., Anjum A.A., Swan P., Sweazea K. (2017). Evaluation of white sesame seed oil on glucose control and biomarkers of hepatic, cardiac, and renal functions in male Sprague-Dawley rats with chemically induced diabetes. J. Med. Food.

[6] Yang J., Wu Y., Li B., Liu L., Ouyang J. (2014). Fourier transform near infrared spectroscopy in the authentication and adulteration of sesame oil. J. Chin. Cereals Oils Assoc..

[7] Moore J.C., Spink J., Lipp M. (2012). Development and application of a database of food ingredient fraud and economically motivated adulteration from 1980 to 2010. J. Food Sci..

[8] Yuan Y.-Y., Wang S.-T., Wang J.-Z., Cheng Q., Wu X.-J., Kong D.-M. (2020). Rapid detection of the authenticity and adulteration of sesame oil using excitation-emission matrix fluorescence and chemometric methods. Food Control.

[9] Karami H., Rasekh M., Mirzaee-Ghaleh E. (2020). Qualitative analysis of edible oil oxidation using an olfactory machine. J. Food Meas. Charact..

[10] Soltani Firouz M., Rashvand M., Omid M. (2021). Rapid identification and quantification of sesame oils adulteration using low frequency dielectric spectroscopy combined with chemometrics. LWT.

[11] Zhao Z., Wu X., Liu H. (2022). Vision transformer for quality identification of sesame oil with stereoscopic fluorescence spectrum image. LWT.

[12] Filoda P.F., Fetter L.F., Fornasier F., Schneider R.d.C.d.S., Helfer G.A., Tischer B., Teichmann A., da Costa A.B. (2019). Fast methodology for identification of olive oil adulterated with a mix of different vegetable oils. Food Anal. Methods.

[13] Jiang J., Dou X., Zhang L., Mao J., Yu L., Ma F., Li P. (2020). Rapid authentication of sesame oil using ion mobility spectrometry and chemometrics. Oil Crop Sci..

[14] Zhang L., Huang X., Li P., Na W., Jiang J., Mao J., Ding X., Zhang Q. (2017). Multivariate adulteration detection for sesame oil. Chemom. Intell. Lab. Syst..

[15] Lyu W., Yuan B., Liu S., Simon J.E., Wu Q. (2021). Assessment of lemon juice quality and adulteration by ultra-high performance liquid chromatography/triple quadrupole mass spectrometry with interactive and interpretable machine learning. J. Food Drug Anal..

[16] Roy M., Doddappa M., Yadav B.K., Shanmugasundaram S. (2022). A novel technique for detection of vanaspati (hydrogenated fat) in cow ghee (clarified butter fat) using flash gas chromatography electronic nose combined with chemometrics. J. Food Process. Preserv..

[17] Xing C., Yuan X., Wu X., Shao X., Yuan J., Yan W. (2019). Chemometric classification and quantification of sesame oil adulterated with other vegetable oils based on fatty acids composition by gas chromatography. LWT.

[18] Luo Q., Chen Y., Xu Q., Yu Y., Zheng X. (2018). Near-infrared-based identification of sesame oil authenticity. IOP Conf. Ser. Mater. Sci. Eng..

[19] Giacomino A., Inaudi P., Silletta G., Diana A., Bertinetti S., Gaggero E., Malandrino M., Stilo F., Abollino O. (2023). Analytical methods for the characterization of vegetable oils. Molecules.

[20] Conrado J.A.M., Sequinel R., Dias B.C., Silvestre M., Batista A.D., Petruci J.F.d.S. (2021). Chemical QR Code: A simple and disposable paper-based optoelectronic nose for the identification of olive oil aroma. Food Chem..

[21] Majchrzak T., Wojnowski W., Dymerski T., Gębicki J., Namieśnik J. (2018). Electronic noses in classification and quality control of edible oils: A review. Food Chem..

[22] Huang Z.-M., Xin J.-X., Sun S.-S., Li Y., Wei D.-X., Zhu J., Wang X.-L., Wang J., Yao Y.-F. (2021). Rapid identification of adulteration in edible vegetable oils based on low-field nuclear magnetic resonance relaxation fingerprints. Foods.

[23] Sysoev V.V., Strelcov E., Kolmakov A., Carpenter M.A., Mathur S., Kolmakov A. (2013). Multisensor micro-arrays based on metal oxide nanowires for electronic nose applications. Metal Oxide Nanomaterials for Chemical Sensors.

[24] Boeker P. (2014). On ‘electronic nose’ methodology. Sens. Actuators B Chem..

[25] Rasekh M., Karami H., Wilson A.D., Gancarz M. (2021). Classification and identification of essential oils from herbs and fruits based on a MOS electronic-nose technology. Chemosensors.

[26] Röck F., Barsan N., Weimar U. (2008). Electronic nose: Current status and future trends. Chem. Rev..

[27] Rasekh M., Karami H., Wilson A.D., Gancarz M. (2021). Performance analysis of MAU-9 electronic-nose MOS sensor array components and ANN classification methods for discrimination of herb and fruit essential oils. Chemosensors.

[28] Gutierrez-Osuna R., Nagle H.T., Kermani B., Schiffman S.S., Pearce T.C., Schiffman S.S., Nagle H.T., Gardner J.W. (2002). Chapter 5—Signal Conditioning and Preprocessing. Handbook of Machine Olfaction: Electronic Nose Technology.

[29] Tang X., Yu Z. (2020). Rapid evaluation of chicken meat freshness using gas sensor array and signal analysis considering total volatile basic nitrogen. Int. J. Food Prop..

[30] Karami H., Rasekh M., Mirzaee-Ghaleh E. (2020). Comparison of chemometrics and AOCS official methods for predicting the shelf life of edible oil. Chemom. Intell. Lab. Syst..

[31] Jolliffe I.T., Cadima J. (2016). Principal component analysis: A review and recent developments. Philos. Trans. R. Soc. A.

[32] Abu-Khalaf N., Masoud W. (2022). Electronic nose for differentiation and quantification of yeast species in white fresh soft cheese. Appl. Bionics Biomech..

[33] Puertas G., Cazón P., Vázquez M. (2023). A quick method for fraud detection in egg labels based on egg centrifugation plasma. Food Chem..

[34] Latif G., Ben Brahim G., Iskandar D.N.F.A., Bashar A., Alghazo J. (2022). Glioma tumors’ classification using deep-neural-network-based features with SVM classifier. Diagnostics.

[35] Ding W., Zhang Y., Kou L., Jurick W.M. (2015). Electronic nose application for the determination of penicillin G in Saanen goat milk with Fisher discriminate and multilayer perceptron neural network analyses. J. Food Process. Preserv..

[36] Schmidhuber J. (2015). Deep learning in neural networks: An overview. Neural Netw..

[37] Savage G.P., McNeil D.L. (1998). Chemical composition of hazelnuts (*Corylus avellana* L.) grown in New Zealand. Int. J. Food Sci. Nutr..

[38] Mohammadian N., Ziaiifar A.M., Mirzaee-Ghaleh E., Kashaninejad M., Karami H. (2023). Nondestructive technique for identifying adulteration and additives in lemon juice based on analyzing volatile organic compounds (VOCs). Processes.

[39] Zhao M., Chen B., Ferranti P. (2023). Corn Oil. Sustainable Food Science—A Comprehensive Approach.

[40] Kong W., Baeyens J., De Winter K., Urrutia A.R., Degrève J., Zhang H. (2019). An energy-friendly alternative in the large-scale production of soybean oil. J. Environ. Manag..

[41] Fan S., Chang J., Zong Y., Hu G., Jia J. (2018). GC-MS Analysis of the composition of the essential oil from *Dendranthema indicum* Var. Aromaticum using three extraction methods and two columns. Molecules.

[42] Zunin P., Boggia R., Salvadeo P., Evangelisti F. (2005). Geographical traceability of West Liguria extra virgin olive oils by the analysis of volatile terpenoid hydrocarbons. J. Chromatogr. A.

[43] Bougrini M., Tahri K., Haddi Z., Saidi T., El Bari N., Bouchikhi B. (2014). Detection of adulteration in argan oil by using an electronic nose and a voltammetric electronic tongue. J. Sens..

[44] Lerma-García M.J., Simó-Alfonso E.F., Bendini A., Cerretani L. (2009). Metal oxide semiconductor sensors for monitoring of oxidative status evolution and sensory analysis of virgin olive oils with different phenolic content. Food Chem..

[45] Xu L., Yu X., Liu L., Zhang R. (2016). A Novel method for qualitative analysis of edible oil oxidation using an electronic nose. Food Chem..

[46] Cerrato Oliveros M.C., Pérez Pavón J.L., García Pinto C., Fernández Laespada M.E., Moreno Cordero B., Forina M. (2002). Electronic nose based on metal oxide semiconductor sensors as a fast alternative for the detection of adulteration of virgin olive oils. Anal. Chim. Acta.

[47] Roy M., Yadav B.K. (2022). Electronic nose for detection of food adulteration: A review. J. Food Sci. Technol..

[48] Men H., Chen D., Zhang X., Liu J., Ning K. (2014). Data fusion of electronic nose and electronic tongue for detection of mixed edible-oil. J. Sens..

[49] Marina A.M., Che Man Y.B., Amin I. (2010). Use of the SAW sensor electronic nose for detecting the adulteration of virgin coconut oil with RBD palm kernel olein. J. Am. Oil Chem. Soc..

[50] Hong E.J., Park S.J., Choi J.Y., Noh B.S. (2011). Discrimination of palm olein oil and palm stearin oil mixtures using a mass spectrometry based electronic nose. Food Sci. Biotechnol..

[51] Man Y.C., Gan H.L., NorAini I., Nazimah S.A.H., Tan C.P. (2005). Detection of lard adulteration in RBD palm olein using an electronic nose. Food Chem..

[52] Wei X., Shao X., Wei Y., Cheong L., Pan L., Tu K. (2018). Rapid detection of adulterated peony seed oil by electronic nose. J. Food Sci. Technol..

